# 

**Published:** 1891-03

**Authors:** 


					﻿Entered at the Post Office, at Austin, Texas, as Second Class Mail Matter


Vol. VI
MARCH,1891.
No.9
DANIEL'S
MEDICAL
JOURNAL
F. E. DANIEL., M. D., Editor and Publisher, AUSTIN, TEXAS.
Northern office, 1217 Filbert Street, Philadelphia.

Steam Book and Jor Printer, Austin, Texas
				

